# Panacea for the nanoplastic surge in Africa: A state-of-the-art review

**DOI:** 10.1016/j.heliyon.2022.e11562

**Published:** 2022-11-14

**Authors:** Emmanuel Sunday Okeke, Onome Ejeromedoghene, Charles Izuma Addey, Edidiong Okokon Atakpa, Semiu Folaniyi Bello, Timothy Prince Chidike Ezeorba, Kingsley Ikechukwu Chukwudozie, Charles Obinwanne Okoye

**Affiliations:** a Institute of Environmental Health and Ecological Security, School of Environment & Safety Engineering, Jiangsu University, Zhenjiang 212013, China; b Department of Biochemistry, Faculty of Biological Sciences, University of Nigeria, Nsukka 41000, Enugu State, Nigeria; c Natural Science Unit, School of General Studies, University of Nigeria, Nsukka 41000, Enugu State, Nigeria; d School of Chemistry and Chemical Engineering, Southeast University, Jiangning District, Nanjing, Jiangsu Province, 211198, PR China; e College of Ocean and Earth Sciences, Xiamen University, China; f Ocean College, Zhejiang University, Zhoushan, 316021, Zhejiang, China; g Department of Animal & Environmental Biology, University of Uyo, Akwa Ibom State, 1017, Nigeria; h Department of Animal Genetics, Breeding and Reproduction, College of Animal Science, South China Agricultural University, Guangzhou 510642, China; i Department of Microbiology, University of Nigeria, Nsukka 410001, Nigeria; j Biofuels Institute, School of Environment & Safety Engineering, Jiangsu University, Zhenjiang 212013, China; k Department of Zoology & Environmental Biology, University of Nigeria, Nsukka 410001, Nigeria; l Key Laboratory of Intelligent Agricultural Machinery Equipment, Jiangsu University, Zhenjiang 212013, China; m Organization of African Academic Doctor (OAAD), Off Kamiti Road, P. O. Box 25305000100, Nairobi, Kenya

**Keywords:** Microplastics, Nanoplastics, Environmental hazards, Plastics, Paradigm shifts, Africa

## Abstract

Africa is a large continent ranked amongst the top consumer of plastic materials. However, the improper handling of plastic wastes has resulted in massive pollution of different aspects of the environment (water, soil, sediments, air, food, etc.) within and around the region. The fragmentation and biodegradation of the bulk plastic material into small-sized particles has given rise to microplastic and nanoplastics. Owing to their small sizes, ease of transport, and large surface area, they tend to wreak serious havoc in the environment. Nevertheless, the growing awareness of the pollution problems caused by micro/nanoplastic debris is instrumental towards circumventing its widespread across the ecosystem. This review provides a state-of-the-art information on the prevailing nanoplastic surge across the borders of Africa, the ineffective management policies of plastic wastes, potential environmental hazards, and possible remediation strategies. Additionally, prospective insights into new areas for advanced research were highlighted.

## Highlights

• The paradigm shift from MPs to NPs has been elucidated.
• The production, consumption, and waste generation of NPs in Africa have been discussed.
• The impacts, detection, and remediation strategies for NPs have been exhaustively reviewed.
• Problems facing the management of NPs pollution in Africa have been carefully explained.
• Future prospects on NPs management in Africa have been suggested.

## Graphical abstract

**Figure:**
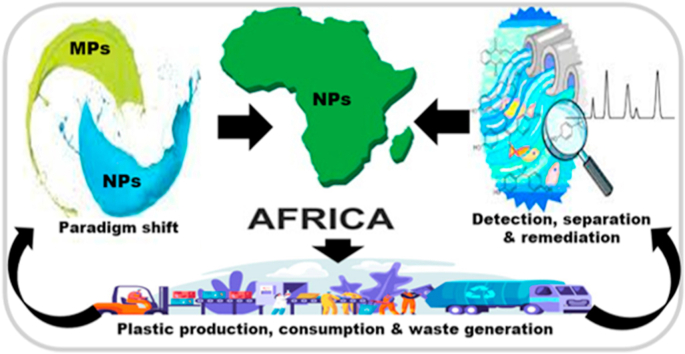

## 1 Introduction

Plastics have largely superseded stone, wood, and metal due to their outstanding physical and chemical qualities and low cost. In many ways, plastic is a valuable resource, yet it is also an unwanted and unsustainable waste of a particular resource. The indiscriminate introduction and circulation of plastics and plastic containing products including plastics cans used in pharmaceuticals in different parts of the world result in pollution [1, 2, 3, 4, 5]. Plastic pollution has become one of the world's most critical environmental issues due to poor handling and the inability of plastic fragments to degrade, including microplastics and nanoplastics [6]. Microplastics (MPs) and nanoplastics (NPs) are formed when plastic fragments are exposed to ultraviolet light, weathering, and biodegradation [7]. Because of their unique characteristics, such as hydrophobicity, surface charges, more extended molecular chain arrangement, large surface area, variety of size, shape, and color, the invention of MPs and NPs have presented additional challenges and research opportunities for the scientific community [8, 9, 10]. NPs are defined as polymers with a size of less than a micron. The size of NPs is specified as either 100 nm or 1000 nm in one dimension, although the scientific definition is still up for controversy [11]. NPs pollution has recently gained a lot of attention as a new persistent environmental problem. These plastic particles are found in abundance in the world's terrestrial environments, freshwaters, oceans, and seas, as well as in the water column and sediments that extend to the deep sea [7]. The accumulation of NPs in the environment is associated with adverse effects on ecosystem functions and human health [12]. Besides, their occurrence leads to several environmental issues through bioaccumulation and toxicity [13]. The adverse biological effects of NPs have been found on various organisms, from bacteria and alga to plants and animals [14].

Plastic pollution and its adverse effects have been reported for over 50 decades, resulting in MPs-based research in ecosystems; however, there is limited focus on NPs in the environment [15]. Although managing plastic pollution is challenging, it is essential to control the utilization of plastic products containing NPs and their release into the ecosystem by introducing new rules and regulations [16]. As a result, environmentalists, organizations, and governments now recognize the need to constantly explore the surge of plastic pollution in the atmospheric, aquatic and terrestrial environment [17]. According to recent studies, NPs pollution research in Africa is severely hampered by a lack of scientific facilities, limiting research activities to options for external collaboration [18]. Despite Africa's top position in unmanaged plastic waste, there is a lack of data on the prevalence of these plastic particles in its ecosystems and their interactions with other pollutants [19]. Although MPs and NPs pollution have been reported worldwide, specific data from Africa is required for reliable risk assessment and policy development [20]. Since NPs will continue to form from MPs due to multiple processes of physical fragmentation and chemical transformations, given the severity of the present plastic pollution, their respective occurrence and implications require detailed and progressive investigation.

Holistically, this review utilized the extraction of relevant manuscripts that critically reported on plastics in Africa and beyond to highlight the current status of plastic production and consumption in Africa, emphasizing the paradigm shifts from MPs to NPs, and discussing the analytical methods for detection and removal of micro/nano plastics from different environmental compartments. In addition, the manuscript adjudges the existing information on the toxicity of NPs to organisms and their implications in Africa and deliberates on the current gaps by presenting a state-of-the-art worldview of NPs surge for future research prospects.

## 2 The paradigm shifts from microplastics to nanoplastics

Generally, the fragments derived from the degradation of plastic particles are usually very small in size; hence they are referred to as microplastics (<5 mm in diameter and 0.1 μm−5 mm in size). However, recent investigations have shown that there are some even smaller plastic fragments (<100 nm in diameter and 0.001–0.1 μm in size) that can penetrate the tissues and organs of organisms, thereby wreaking serious havoc in the ecosystem [21]. The chief compositions of microplastic particles are majorly heterogeneous and are available as a variable proportion of the most common polymer components like polystyrene (PS) and polyethylene (PE), and polyethylene terephthalate (PET) [22, 23]. Reports on the macro-abundance of microplastic in environmental samples have grown consistently with serious concerns about its effect on environmental pollution and human health implications [24]. The presence of these small particles in the environment can alter the regular functioning of ecosystems, altering various life forms. These impacts are widely attributed to the leaching and adsorption of toxic pollutants from plastic contaminants [25]. With the tremendous concern of microplastics, it is still puzzling to ascertain if NPs are an extension of the microplastic problem, bearing in mind that the smaller particle sizes may outrightly trigger different interactions modes with the biota and may likely affect their fate and transport pathways. While the awareness of the inherent dangers of plastic pollution to all forms of life, NPs are alleged to be worse due to their nanosized distribution, high surface area, and surface charge [26].

Although there is not much information to carry out an adequate risk assessment of NPs, some key knowledge gaps must be filled in terms of NPs hazards and exposure [27]. Nanoplastics are suspected to be released into the environment directly from commercial polymer-based personal care products and by the subsequent breakdown and distribution of larger pieces of plastic litter [28]. Recently, the number of environmental science studies on NPs has been increasing progressively since the mid-last decade, with polystyrene nanoparticles (PSNP) as the major NPs of concern [29, 30]. In a short communication, Lambert and Wagner [31] hypothesize that NPs are formed during the degradation of a disposable PS coffee cup lid, and the increase in concentration over time is measurable. Meanwhile, Ekvall et al. [32] asserted that the daily usage of PS products could instigate the mechanical breakdown of PSNP with different sizes and surface modifications and negatively affect wildlife. For example, exposure to PSNP (50 μg/mL) can dysregulate lipid metabolism in murine macrophages in an *in vitro* study [33]. The micro-injection and bioaccumulation of PSNP (20 nm) caused oxidative DNA damage in the brain tissue of zebrafish embryos [34]. In the investigations of Lian et al. [35], 0.01–10 mg/mL of PSNP showed no distinct effect on the seed germination rate of wheat (*Triticum aestivum* L.) but could reduce the shoot to root biomass ratio of the seedling. Besides, in a nanotoxicological and transcriptome investigation, Lian et al. [36] deduced that PSNPs could significantly shape wheat plant gene expression patterns in a tissue-specific manner.

Furthermore, PE nanoplastics (PENPs) and PET nanoplastics (PETNPs) are other groups of polymer-derived nanoplastics that are majorly used in drinking bottles and packaging materials, and their size and surface coating-dependent toxicological potentials have not received much attention over the years [37]. Nevertheless, few authors have endeavored to investigate the impact of these NPs pollutants in the environment. For example, the exposure of PENPs to zebra embryos resulted in severe pericardial edema as well as a significant decrease in cardiac output and blood flow velocity. NPs could also inhibit the subintestinal angiogenesis of transgenic zebrafish embryos line Tg (fli-1: EGFP), disturbing cardiovascular formation and development [38]. The PETNPs prepared by Magrì et al. [39] was reported to be internalized in endolysosomes, where they demonstrated intracellular biopersistence and long-term stability in a simulated lysosomal environment. Interestingly, the PETNPs also displayed a high tendency to migrate via the gut barrier, with an uncertain long-term effect on health as well as potentially transporting dispersed chemicals mediated by the nanopollutants when monitored on a model of the intestinal epithelium.

## 3 Plastic production, consumption, and waste generation in Africa

**Figure 1:**
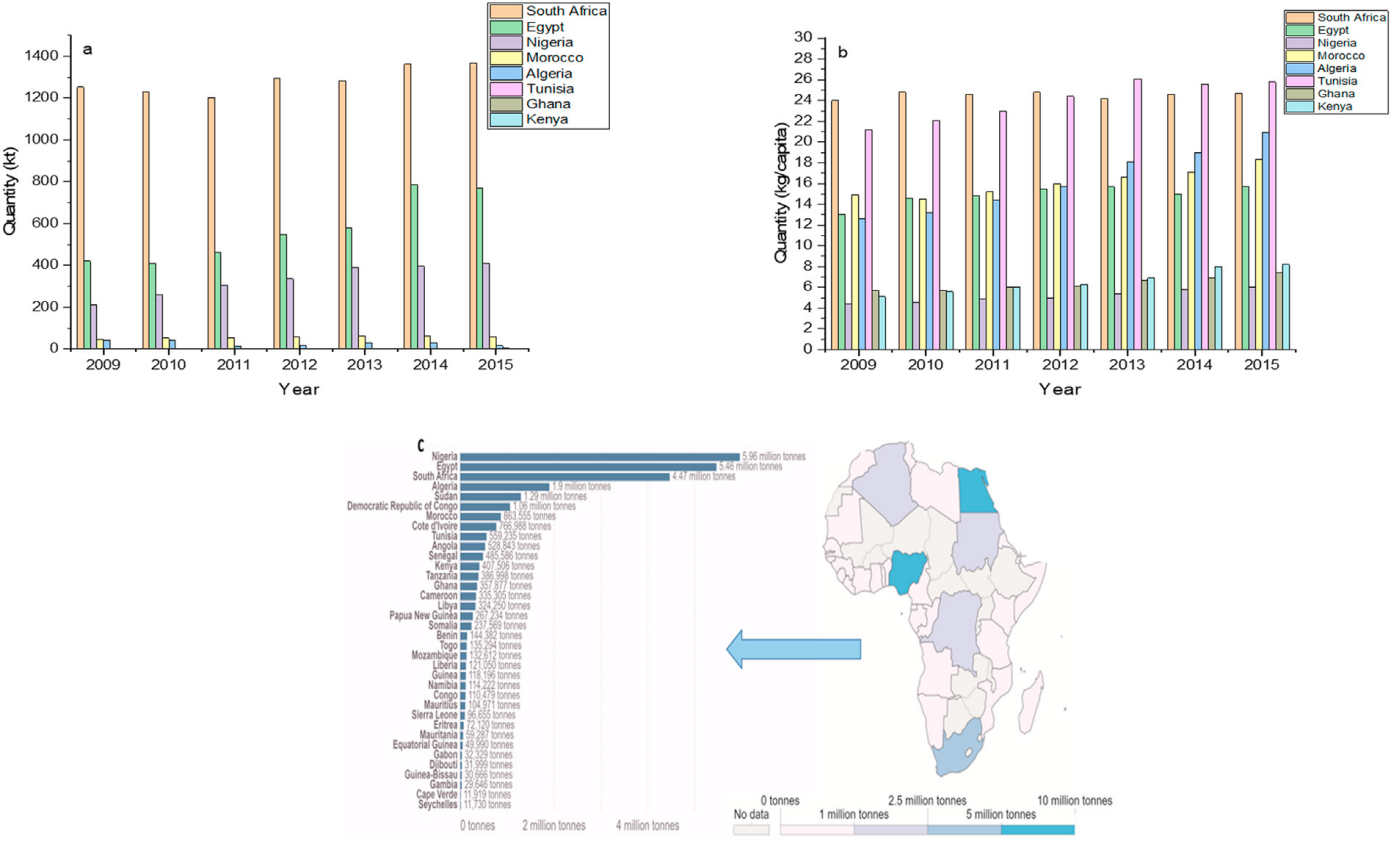
Plastics production (**a**), consumption (**b**), and waste generation (**c**) in top African countries from 2009-2015 adapted from Babayemi et al. (2019), and Ritchie and Roser (2018).

Plastic production has steadily increased over the past seven decades, ranging from 1.5 million metric tonnes (Mt) in the 1950s to 359 million Mt presently, and its consumption is considered one of the primary sources of waste [40]. Plastics are prevalent in Africa and worldwide due to their ease of use, thin weight, flexibility, and affordability. The global viewpoint is that the production and consumption of plastics will double in the next two decades and can only increase if alternative solutions, including waste management policies and banning of plastic bags, are not implemented soon [41, 42, 43]. The rapidly increasing demand for plastic products across Africa has established itself as a significant player in the international plastics and packaging business, with considerable demand for plastic goods. Africa's plastic sector is experiencing rapid expansion as the continent's demand for plastic products and machinery continues to rise. Plastics production machinery (PPM) and plastics material resins (PMR) are two of the categories recognized as potential opportunities for foreign corporations in most African countries [44]. Most African countries resolved to import plastics from Europe and Asia in more significant quantities as raw materials than finished goods, indicating that these African countries have substantial plastic processing and production activities using imported primary polymers. A recent study suggests that most African countries will increase their imports by 2030 [45]. This could lead to the accumulation of billions of tonnes of plastic particles in terrestrial and aquatic ecosystems soon, consequent on the lack of a proper waste management plan and strategy [1, 3, 4, 46, 47]. The increase in the production of plastic is linked to significant consumption and waste generation [15]. Information on plastic consumption has also relied on waste generation data per capita plastic particles from municipal solid wastes [3]. Plastic consumption in the 54 African countries is estimated at approximately 19.71 Mt based on per capita plastic consumption of 16 kg/year, with eight countries, including South Africa, Egypt, Nigeria, Morocco, Algeria, Tunisia, Ghana, and Kenya, having the highest rankings of plastic production and consumption over the past decade (Fig. 1a and b). However, there is a likelihood that plastic consumption in Africa will increase to 41.57 Mt by 2030 [42]. Plastic production and consumption have resulted in a considerable volume of plastic waste globally, negatively impacting human and animal health, and is currently at the top of the international waste management agenda [42, 48]. Plastic products are present in all environmental compartments as plastic particles, derivable from single-use [49, 50].

Plastic waste is a huge problem that needs to be addressed immediately since the production and consumption of plastics are increasing, resulting in increased plastic waste generation across Africa [41]. However, plastic waste management is insufficient, particularly in African countries, and most plastic particles end up in the aquatic ecosystem [17]. Presently, Nigeria, Egypt, and South Africa rank high in the global plastic waste generation of 5.96, 5.46, and 4.47 million tonnes annually (Figure 1c) [51]. Moreover, Nigeria, Cameroon, Algeria, and Tanzania were among the top 20 countries with riverine discharges of plastic wastes into the ocean, contributing to about 84,792 tonnes of MPs and NPs discharged into the Mediterranean Sea from the 3.3 million tonnes of mismanaged plastic waste generated in the Nile River catchment in 2017 [52]. Increased plastic production, consumption, and waste generation are the leading causes of NPs pollution in Africa [43]. Therefore, we must understand the linkage of plastic production, consumption, and waste generation to comprehend the volume of plastics inflow in the African environment (Figure 1). This is essential for determining the surge of NPs pollution, which has become a ubiquitous and emerging environmental and public health concern in Africa, and adopting the most effective reduction strategies [43, 53].

## 4 Analytical methods for detection of micro/nano plastics from different environmental matrices

**Figure 2:**
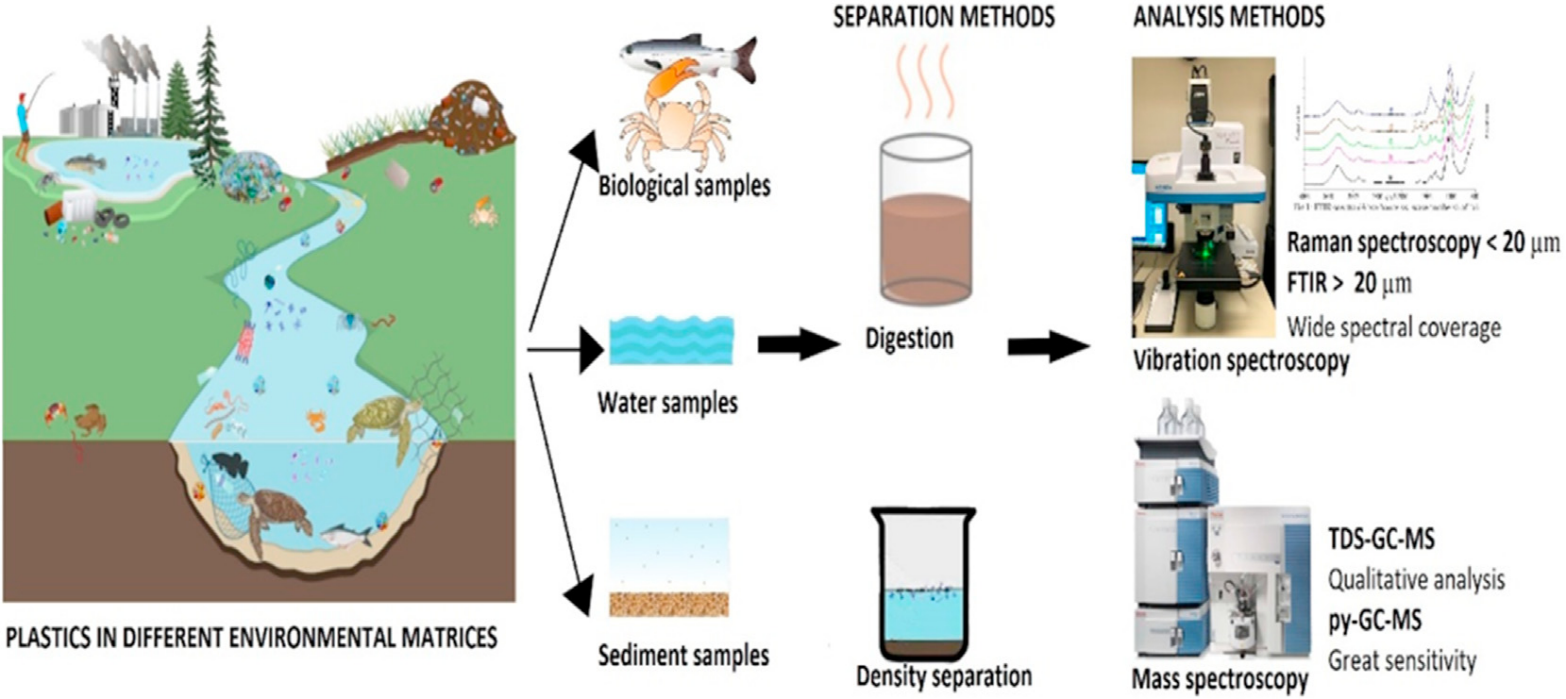
Nanoplastics separation and detection in different environmental matrices.

Micro (nanoplastics) are polymers created either purposefully or through the fragmentation of larger polymers [54]. The different micro (nanoplastics) polymer types break into tiny particles that enter and infest the aquatic and terrestrial environment [55, 56, 57]. Macro- and mesoplastic fragments (those larger than 5 mm in diameter) are distinguished from microplastics (those less than 5 mm) and nanoplastics (within the range of 1 nm to 1 mm) [58]. In recent decades, they have risen to the status of a serious global environmental problem [9], and recent scientific research has revealed that these particles may be found all over the world, even in areas that were previously assumed to be uncontaminated [57]. Nowadays, it has increasingly become routine to conduct micro/nanoplastics analysis on samples from biotic and abiotic environmental compartments [54, 59, 60, 61]. Thus, it is necessary to develop reliable procedures for identifying these microscopic and nanometric-sized particles. Here, we cover the present and possible detection methods used in the analysis of microplastic, as well as their merits and limitations. From physical to chemical procedures, the most appropriate techniques are reviewed. Even though microscopical techniques are among the widely used methods for NPs detection [57], they have the drawback of producing incomplete results when analyzing small particles. The combination of chemical analysis (spectroscopy) and recently presented alternative methodologies has, for the time being, been successful in overcoming this limitation. Current issues in the detection of NPs might be solved by developing new analytical equipment that works in conjunction with one another or with traditional and creative microscopy techniques (Figure 2).

### 4.1 Analytical methods of micro/nanoplastics detection

#### 4.1.1 Fourier transform infrared (FTIR) spectroscopy

Infrared spectroscopy, also known as IR spectroscopy, is a spectroscopic method commonly employed in material classification. After being absorbed by a particle, an infrared photon causes the particle to transition from its basic vibrational state into an excited state.

FTIR is now the most widely utilized technology for identifying microplastic components due to its non-destructive sampling, easy identification process, and high accuracy (Table 1) [59,[62], [63], [64]]. This approach may not only detect microplastics but also distinguish particular types of polymers by comparing the infrared spectra of sample particles with the standard spectra of a polymer in the spectral library, offering information for the origins or input of samples [59, 65]. Traditional infrared spectroscopy, on the other hand, is time-consuming and involves manual sorting of sample particles in advance to define the target to be studied, and microplastics in tiny quantities and with small particle size may be overlooked [66].

**Table 1:** Advantages and limitations of analytical methods of detecting nanoplastics.

Detection Method	Advantages	Limitations	Technology Readiness Level (TRL) Scale	References
FT-IR Spectroscopy	• Small microplastic detection (>20μm). • Non-destructive method and easy to operate. • No false or negative reading. • Has sufficient spectrograms, which enhances the resolution.	• Expensive instrumentation. • Sample thickness insensitivity. • Wavelength radiation. • Time-consuming	5–7	[57, 65, 67, 68, 69, 70]
Raman Spectroscopy	• Wider spectral Coverage. • RS is useful for very tiny plastic sizes (<20 μm). • Better resolution. • No false or negative reading. • Lower water interference. • Non-destructive method.	• Very expensive. • Fluorescence interference. • Laser-induced degradation. • Time-consuming.	5–7	[57, 71, 72, 73]
Thermal analysis	• High sensitivity and efficiency. • Detection of complex samples. • Not time-consuming. • Can characterize low solubility microplastics.	• Destructive method. • Just one evaluation can be done per run.	3–5	[57, 65]

TRL is assigned on a scale of 1–9, showing development status across the entire range of emerging technologies. 1 indicates the least level, while 9 is the most advanced technology [74].

Reflectance [75], Transmission [76] are some of the approaches used in FTIR. Every analysis involves the excitation of microplastics samples, which detects certain vibrations that allow the generation of spectra with a signature range. This spectrum indicates the feature of the substance, which may be determined by comparing it to the spectrums of other materials studied previously. FTIR may be used to investigate larger particles (>500 nm), but the micro-FTIR (μ-FTIR) is required for small particles because it allows for simultaneous viewing, mapping, and collecting of spectra [57]. In both ATR and reflectance modes, it is possible to do this procedure. For example, Jung et al. (2018) presented a conclusive confirmation of ATR FTIR to detect swallowed polymer composite types in sea turtles. ATR allows for the acquisition of high-quality spectra; however, because of refractive error, uneven particle surfaces might create interference with the analysis process [77]. Only samples with specific qualities may be evaluated; otherwise, the signal cannot be recognized as valid. The lateral resolution of the instrument is also restricted to the diffraction range, and samples with dimensions less than 20 m are not discernible [65]. FTIR has been widely utilized in microplastics research to locate and detect microplastics in a variety of environments, including sediment [63, 77], aquatic fauna [65, 78, 79], surface water [60], and food [60, 77, 80, 81]. IR signals may be collected with a great spatial resolution with this technology, and it is particularly beneficial for the characterization of complicated materials due to its versatility. Among other things, Zhang et al. [82] conducted an FTIR analysis on microplastics in sediment samples from twenty-eight locations within Sishili Bay, detecting 8 polymer types such as polyethylene (PE), polymethyl methacrylate (PMMA), polypropylene (PP), polystyrene (PS), polyurethane (PU), and rayon.

Atomic force microscopy (AFM) is used to scan the surfaces of particles and subsequently create images of their height; however, the technology does not have the capability of determining the molecular composition of the materials being scanned [57]. Researchers created the AFM-IR technology as a result of combining AFM and infrared spectroscopy. This approach is frequently used to collect infrared absorption spectrum with a spatial resolution of about 50–100 nm, then utilized to characterize the sample [83]. When a sample is inserted into the AFM-IR microscope using a micro-lever, one side-end is attached to the support and another end connected to a sharp tip. The measurement of the undetectable motions of the sample is derived using an IR laser beam. The sample particle absorbs light, resulting in a little expansion and, as a result, causes the micro-lever to deflect, which results in the generation of IR signals. Thermal expansion of the samples caused by the absorption of light by the sample creates thermal energy and generates an impulse on the AFM, causing the cantilever to oscillate [83]. Merzel et al. [84] employed a combination of the AFM-IR approach and photothermal infrared spectroscopy to detect the beads in the mussel siphons. In certain cases, the approach has been effectively employed to investigate samples that had been previously identified as being infested with plastic particles. According to some researchers, characterizing unidentified samples may expose technical shortcomings because it is difficult and takes a lot of time to scan and detect NPs in an unidentified sample [57].

#### 4.1.2 Raman spectroscopy (RS)

Raman spectroscopy (RS) is a method that works on the interplay between materials and radiation. A laser beam is used in this approach, which engages with the vibrational movements of the molecules and produces the re-emission of light at unique wavelengths. Raman spectroscopy is yet another chemical analysis method that may be used to detect plastics in various environmental compartments. Van Cauwenberghe et al. [85] found plastic debris in the μm-range in bottom sediments collected from different sampling sites in the marine environment with a depth ranging from about 1000 to 5000 m. Cole et al. (2013) discovered that 13 species of zooplankton could ingest about 1.7–30.6 μm of polystyrene beads. Uptake rate varied based on species type, life stage, and bead size. The Raman micro-spectroscopy technique was used to detect and quantify the particles, which allowed the investigation of particles in μm-range. Traditional Raman spectroscopy often identifies microplastics bigger than 10 μm, but micro-Raman spectroscopy (μ-Raman spectroscopy) analyzes microplastics as small as 1 μm. This coupling of Raman spectroscopy and optical microscopy allows for visually picking the particular material area to be studied for further investigation. Due to this, it may be applied in a wide range of scientific disciplines, with the most notable uses being in the fields of environmental science, materials science, and biology [57, 86]. The technique has recently been applied to detect microplastics in bottled water and the analysis of the release of microplastics from bottled water containers in recent years [87]. Other uses of RS, such as the detection of microplastics in diverse environmental compartments such as seawater [88], sediment [89, 90, 91], and aquatic faunas [92].

#### 4.1.3 Thermal analysis (TA)

Besides the FTIR and RS methods discussed above, the thermal analysis method has also been used for detecting microplastics [57, 65]. It includes Elemental analysis (EA), thermogravimetric analysis coupled with differential scanning calorimetry (TGA-DSC), and gas chromatography-mass spectrometry (GC-MS). The thermal stability of polymer samples is used to determine the changes in polymers' physical and chemical properties. Polymers absorb a significant amount of heat as they transition from one phase to another (i.e., gas, liquid), resulting in an endothermic peak. The TGA-DSC technique is a reliable method for determining the thermal characteristics of polymeric materials. Majewsky et al. (2016) showed that the TGA-DSC technique could distinguish between a combination of PP and PE, due to the considerable difference in the temperatures of their endothermic peaks (164 ± 1 °C and 101 ± 2 °C, respectively). However, it is unable to distinguish between polyamide (PA), polyethylene terephthalate (PET), polyvinyl chloride (PVC), and polyurethane (PU) since their peak temperatures are comparable and their signals overlap. Thermal desorption gas chromatography-mass spectrometry (TDS-GC-MS) and Pyrolysis gas chromatography-mass spectrometry (Pyr-GC-MS) are two GC-MS technologies utilized for the identification of microplastics. Microplastics may be analyzed using the Pyr-GC-MS approach, which can assess their chemical characteristics by studying their pyrolysis components [65]. Using acquired thermal spectra compared to reference thermal spectra obtained from known samples, the polymer content may be evaluated and validated using thermal spectroscopy [93].

To extract organic additives over a long time, the samples were first put at a low temperature and then heated continually, while microplastics were degraded at a certain temperature. There is no need to add solvents during the process, which eliminates the possibility of background contamination. It does, however, have the potential to misjudge the kinds of microplastics since various polymers might yield pyrolysis products that are identical to one another. Apart from that, because it can only analyze 0.5 mg of materials at a time, the Pyr-GC-MS approach is not ideal for investigating composite and diverse samples, such as those found in biological samples. In addition, because it requires a significant amount of time and allows only one particle to be evaluated at a time, it is unfit for use in the analysis of standard monitoring and large-scale sampling [94]. TDS-GC-MS requires a minimum of 100 mg of samples, but Pry-GC-MS requires just a few milligrams, and its identification findings are not influenced by degradation due to heating [94]. This approach is steady and appears to be acceptable for examining environmental samples, but its applicability and practicability require more investigation and verification [65].

Thermal analysis may be utilized to determine the polymer content of microplastics, the presence of inorganic or organic additives, and the quality of the additives in samples. Nonetheless, these procedures are destructive, meaning they cannot be utilized for further examination of materials after they have been processed (Table 1) [57, 65, 95]. Thermal analysis techniques can only provide information on the overall mass fraction of polymers; they cannot give explicit details on the size distribution and number of microplastics present in samples. Many constraints remain in the use of thermal analysis to detect microplastics; therefore, there is needed for further investigation into their applicability and feasibility.

## 5 Toxic effects of nanoplastics on organisms

The gradual paradigm shift from MPs to NPs comes with its toxic shifts [52]. This drastic shift is accompanied by a hazardous threat to the environment and the whole ecosystem [96]. Research has shown that NPs could be ingested by an organism with possible accumulation in various tissues and/or organs of these organisms. MPs find their way into the circulatory system via enteric tissues leading to numerous toxic effects at both molecular and cellular levels. A study shows that MP was ingested by African ducks in 2 freshwater system, and MPs were found in the feather brushings and feces of seven southern African duck species [97]. MP particles mainly fibers (95%) were detected in offshore fish namely *Trachurus capensis, Etrumeus whiteheadi, Chelidonichthys capensis, Scomber japonicus, Argyrozona, Merluccius paradoxus and Merluccius capensis* from Agulhas Bank in South Africa [98]. Similarly, microfibers and other plastic were detected in macroinvertebrates sediments, and shorebirds (feces and gizzards) from 3 intertidal wetlands of west Africa [99]. Microplastic particles mainly films, fibres and fragments were detected in along the coastal shelf of KwaZulu–Natal, South Africa [100]. Micro-Fourier-transform infrared spectroscopy (μFTIR) showed the presence of MPs particles (fibre and film) found in common West African gastropods (*Melanoides tuberculate and Lanistes varicus*) in Osun River system, Nigeria were made of polyethylene, suggesting that the ingested MPs by th egatropods could be transferred to other organisms at higher trophic level by predation [101]. In a recent study, the effect of virgin or phenanthrene (Phe)-loaded low-density polyethylene (LDPE) fragments in juvenile African catfish (*Clarias gariepinus*) was investigated [102]. In this study, virgin LDPE (50 or 500 μg/L) were preloaded with Phe (10 or 100 μg/L) and were exposed to *Clarias gariepinus* for 96 h [102]. The result showed that Phe exposure caused a significant increase in rate of tissue damage (DTC) in the liver, reduced expression levels of tryptophan hydroxylase 2 (*tph2*) and forkhead box L2 (*foxl2*). While lowering the transcription levels of tph2, exposure to either concentration of virgin MPs enhanced the DTC in the liver and the plasma albumin:globulin ratio [102]. It was discovered that MP modulated the effect of Phe on the DTC in the gill, concentration of cholesterol in the palsma, high-density lipoprotein, total protein, globulin, albumin and altered the transcription levels of *fushi* tarazu-factor 1, 11 *β*-hydroxysteroid dehydrogenase type 2, gonadotropin-releasing hormone, and glycogen stores in the liver [102]. Large predatory sharks including *Carcharinus obscurus* in the eastern coast of South Africa between 1998 and 2000 were entrapped in polypropylene strapping bands leading to underweight, tissue damage of the sharks, and consequent mortality [103]. A recent study, mussel, *Mytilus edulis*, was used as a model organism to assess the ingestion, translocation, and accumulation of MP [104]. Similarly, MPs were found to cause entanglement in South African whales [105]. In this study, initial research revealed that after ingestion, MPs accumulated in the gut. Then, treatments containing seawater and microplastic (3.0 or 9.6 m) were administered to mussels. Microplastic was found in the hemolymph after it was transferred to clean surroundings. Within 3 days, particles moved from the gut to the circulatory system, where they remained for more than 48 days [104]. Upon ingestion, MPs can remain in the gastrointestinal tract, be ingested through feces, or enter bodily tissues via the epithelial lining of the gut (translocation) [104]. Laboratory studies using humans and rodents have revealed that polystyrene [106] and polyvinylchloride [107] particles smaller than 150 μm can translocate from the gastrointestinal cavity to the lymphatic and circulatory systems. Microplastics (1, 10, 15, 100 mg/L) exposed to juvenile Nile Tilapia (*Oreochromis niloticus*) for 15 days resulted in a significant increase in biochemical parameters including glucose, ALP, AST, ALT, creatinine, cholesterol, albumin, total protein, uric acid, and globulin in a dose dependent manner and significant decrease in hematological indices hemoglobin, RBC count, haematocrit, Platelets, mean corpuscular hemoglobin, monocytes and WBC count [108]. These observed toxic effects of NPs on organisms are closely linked with particle size, morphology, composition, surface properties, and aging time (Table 2).

**Table 2:** Toxic effects of nanoplastics on selected experimental organisms.

Animal model	Particle type	Particle Size (nm)	Exposure Concentration	Exposure period	Observed adverse effects	References
Zebrafish (*Danio rerio*)	Polystyrene (PS)	50	1 mg/L	3 days	1.Upregulation of central nervous system genes 2.Significant inhibition of acetylcholinesterase activity 3.Enhancement of neurotoxicity of the dopaminergic system and the central nervous system	[109]
Algae (*Scenedesmus obliquus*)	PS	70	44–1100 mg/L	72 h	1. Inhibition of growth and development in algae 2. Significant decrease in chlorophyll content resulting in altered photosynthesis 3. Observed oxidative damage of algae	Besseling et al. (2014)
Mussel (*Mytilus galloprovincialis*)	PS	110 ± 6.9	0.5–50 mg/L Carbamazepine 6.3 μg/L	96 h	1. *Mytilus galloprovincialis* have high sensitivity to PS nanoparticles even at very low concentrations 2. Alteration of gene expression 3. Significant decrease in enzyme activity 4. Increase in oxidative stress and peroxidation 5. Induced effects on neurotransmission	[110]
Zooplankton (*Tigriopus japonicus*)	PS	50	0.125–25 mg/L	96 h	1. Decreased fecundity after nano plastic exposure 2.Increased malformation and deformity 3. High death rate of larva 4. Exposure to high concentration of the nano plastics lead to death of parents	[111]
Zebrafish (*Danio rerio*)	PS	70	20 mg/L	7 days	1.Nano plastic exposure resulted in local infection as well as lipid accumulation in the liver 2. Potential alteration in metabolites 3. Disruption of energy metabolism and other mechanisms in the liver of the fish	[112]
Zebrafish (*Danio rerio*)	PS	42	5 mg/L	7 days	1. Maternal transfer through accumulation in egg as a result of NPs interaction with the plasma proteins of oocytes 2. Decreased activity of glutathione thereby modulating the antioxidant system	[113]
Oyster gametes (*Crassostrea gigas*)	PS PS-COOH PS-NH_2_	100	0.1–100 mg/L	1–5 h	1. Observed significant production of ROS in a dose-dependent manner due to exposure to PS-COOH but not PS-NH2. 2. Both nanoparticles had less influence on the oocyte after exposure 3. Significant increase in the relative cell size and complexity of the spermatozoa of the oyster 4. The observed toxicity on the two gametes cells could be linked to difference cell membranes	[114]
Mussel hemocytes (*Mytilus galloprovincialis*)	PS-NH_2_	50	1–50 mg/L	30 min	1. Observed decrease in stabilization of the lysosomal membrane 2. significant increase in oxyradical production in hemolymph serum 3. Induction of rapid cellular damage including loss of filopodia and membrane blebbing 4. PS-NH2 protein corona was generated in hemolymph serum	[115]
Crustacean (*Daphnia galeata*)	PS	52	5 mg/L	5 days	1. Reduced survival and inhibition of reproduction in crustaceans. 2. Abnormal embryo development after exposure to PS nano plastics 3. Significant reduction in hatch rate	[116]
Nematode (*Caenorhabditis elegans*)	PS	100 500	1 mg/L	3 days	1.Inhibition of growth and development of nematodes 2. Alteration in locomotor behavior activities 3. Significant induction of oxidative damage with consequent neurotoxicity	[117]
Zebrafish (*Danio rerio*)	PS	50	1 mg/L	120 h	1. Significant inhibition of acetylcholinesterase activity 2. upregulated expression of phototransduction genes (rhodopsin and blue opsin gene) 3. Reduction in larva body length 4. Alteration in locomotor activity of larva	Chen et al. (2017)
*Daphnia pulex*	PS	75	0.1–2 mg/L	48 h	Nanoplastics obviously induced and inhibit the expression of stress defense genes. Growth and reproduction were affected. The clutch time was delayed, offspring numbers of clutches were decreased and the female numbers of per clutch were also decreased.	[118]
Bacterium (*Halomonas alkaliphila*)	PS PS-NH2	50 55	0–320 mg/L	0–2 h	1. Observed growth inhabitation at high concentrations 2. Induction of higher oxidative Stress by the positively charged NPs than the non-charged NPs and consequent interruption of NH4^+^ conversion efficiencies. 3. In addition, the positively charges NPs were observed to be attached to the cell surface with higher toxicity under the effect of electrostatic activity	[119]
Nematode (*Caenorhabditis elegans*)	PS and TiO_2_ nanoparticle	108.2 ± 4.5 10 ± 2	0.01–1 mg/L	Prolonged exposure	1.Combined exposure to PS and TiO_2_ nanoparticle resulted in change the molecular basis of oxidative stress. 2. Observed enhancement of the toxicity of TiO_2_ (intestinal generation of reactive oxygen species and altered locomotion activity) nanoparticle due to the presence of the nano plastic	[120]
Rotifer (*Brachionus plicatilis*)	PS-COOH PS-NH2	40 50	0.5–50 mg/L	24–48hrs	1. PS-NH2 nanoplastics unlike PS-COOH showed a high mortality 2. PS-COOH caused no observable acute toxicity in rotifers.	[121]
*Daphnia pulex*	PS		1.1 mg/L 2 mg/L	1–21 days	1. A significant difference in the expression of the gene encoding the energy-sensing enzyme AMPK a, b and g when compared to among all age groups. 2. Individual vulnerability to nanoplastic pollution is affected by age, which alters important physiological and biochemical processes such cellular energy homeostasis and oxidation in vivo.	[122]
*Dicentrarchus labrax*	Poly (methyl methacrylate) PMMA	45	0–20 mg/L	96 h	1. The level of mRNA transcript was clearly increased. 2. Exposure to the nano plastic led to immune system impairment 3. Altered molecular signalling pathway consequently leading to interference with lipid metabolism	[110]
Acorn barnacles (*Amphibalanus amphitrite*)	PMMA	45	5–25 ppm	24 h	1. Even at low concentrations of nanoplastics, bioaccumulation of the particles was observed in barnacles in a chronic exposure test. 2. Nanoplastics were shown to persist in the bodies of invertebrate throughout the growth stage from Nauplius to juvenile barnacles, suggesting that nanoplastics could have a long-term impact on invertebrate ecosystems.	[123]

**Table 3:** Plastic pollution Policies in some African countries.

Country	Type of intervention	Year announced	Scope of policy	Enforcement	Ref
Mali	Partial ban	2012	A ban on the production, importation, sale and use of nonbiodegradable plastic bags.	Poorly Enforced	[124]
Togo	Partial ban	2011	A ban on the production, importation, possession and commercial use of non-biodegradable plastics.	Poorly Enforced	[124]
Mauritania	Partial ban	2013	A ban on manufacturing, using, and importing plastic bags	Poorly Enforced	[124]
Senegal	Partial ban	2015	Ban on production, importation, possession and distribution of plastic < 3μ	Poorly Enforced	[124]
Cote D'ivore	Partial ban	2013	A ban the production, importation, commercialisation, possession and the use of any nonbiodegradable plastic bags made of lightweight polyethylene, or similar plastic derivates with a thickness of less than 50 μm	Poorly Enforced	[124]
Niger	Partial ban	2013	A ban on the production, importation, trade, usage and stocking of low-density smooth plastic and packaging bags	Poorly Enforced	[124]
The Gambia	Partial ban	2015	A ban on production, importation, sale and use of plastic bags.	Poorly Enforced	[124]
Guinea-Bissau	Partial ban	2013	A ban on the manufacturing, importation, possession and sale of nonbiodegradable plastics	Poorly Enforced	[124]
Burkina Faso	Partial ban	2015	A ban on the use of non-biodegradable plastic packages and plastic bags	Poorly Enforced	[124]
Nigeria	No ban	-	A bill to ban plastic bags is being considered in the house of representative (lower chamber of parliament) as at May 2019	-	[124]
Guinea	No ban	-	No conscious and concerted effort to deal with plastics in the country	-	[124]
Liberia	No ban	-	No conscious and concerted effort to deal with plastics in the country	-	[124]
Sierra Leone	No ban	-	No conscious and concerted effort to deal with plastics in the country	-	[124]

Recently, it was shown that a charged polystyrene (PS) NP (20 nm) was easily absorbed on green algae surface and adversely affected its photosynthetic process through reduction of chlorophyll content [125]. Furthermore, they also showed that scallops' ability to absorb NPs on the surface of these algae was considerably increased. There were easy accumulation of charged PS NPs (40 nm) in the digestive tract of sea urchin, positively charged particles (PS–NH_2_) demonstrated more noticeable toxicity to sea urchins than negatively charged particles (PS–COOH) [126]. PS-NH_2_ NPs have a strong affinity for lipid bilayers on cell membranes, promoting cell absorption via endocytosis. NPs polymer composition and particle size would have a considerable impact on the toxicity of NPs to organisms. Nanoplastics of smaller sizes may be more easily absorbed by bodily tissues and cells. Removing these NPs particles is significantly more challenging, prolonging their exposure time and enhancing the hazards to organisms.

PS and polymethylmethacrylate (PMMA) NPs were employed more extensively and commonly than other NPs. This may be related to the difficulties of synthesizing alternative polymers. Ward and Kach [127], discovered that mussels (*Mytilus edulis*) could ingest PS NPs particles (30 and 100 nm) with resultant accumulation in their digestive glands. The presence of PS NPs significantly increased neurotoxic effects in the central nervous system and dopaminergic system. Furthermore, a recent study showed that PS NPs could be transported along the food chain from algae, through zooplankton to fish, bind to apolipoprotein A-I in fish serum in-vitro preventing them from properly their fat reserves if absorbed via ingestion (affects lipid metabolism) [128]. They further demonstrated that consuming zooplankton that contains nanoparticles has consequences on the fish's feeding behavior in addition to the metabolic effects [128].

There are other thought-provoking studies on the impact of plastic nanoparticles on organisms. The nanoparticles were shown to cause oxidative stress-related effects compared to microplastics [129]. The effect of NPs on the innate immune system of fish was exclusively studied. Degranulation of neutrophil extracellular trap release, primary granules, and oxidative burst activity were reported [130]. Marques-Santos et al. [131] investigated the effect of amino-modified PS particles and found that these particles produced a corona protein when incubated with celomic fluid. Plastic nanoparticles have both biological and toxicological impacts [132]. The impact of nanoparticle exposure to aquatic organisms ranges from reproductive failure and generation of reactive oxygen species [133].

MPs and NPs are mainly basic polymeric materials and additives derived from plastics and chemicals absorbed from various environment compartments. The toxicity of plastics is associated with its component chemicals including solvents, monomers, catalysts, plasticizers and additive dyes, Tetrabrobisphenol A and tetrabromobisphenol A bis(2-hydroxyethyl) ether [134, 135, 136, 137]. Some low molecular weight additives leach out into the water bodies [138]. These additives cause numerous toxicities to the water bodies, soil, air and its inhabitant organisms. Inhalation or ingestion of these additives cause disruptive effects to the endocrine system, hormonal imbalance, reproductive toxicity, metabolic disorders as well as other neurodevelopmental anomalies [138]. Crystallinity is a set of ordered structural links that affects how polymers behave in terms of density, permeability, and swelling [139]. The amount of time MP/NPs spend in the environment affects their crystallinity. The polymer's amorphous region breakdown encourages overall crystallinity and shrinks MP/NP size [140]. Crystallite materialization may change MP/NPs from their counterparts and change their toxicity. It eventually affects surface area, shape, particle size, chemical characteristics such the adsorption of contaminants and additives, and afterwards affects the rate of ingestion [139]. Marine organisms ingest bright colored NPs particles similar to natural foods. Euphausiid, Zooplankton, fish larvae and copepods, ingest predominantly in blue, red, green, and black colored MPs [139]. The shape of NPs/MPs have an influential impact on the transport of pollutant in the environment [141]. For atmospheric transport, films are thin and have a larger surface area than fragments of the same mass [142]. The size of MP/NPs in the marine and aquatic ecosystem influences their availability to pelagic and benthic habitats [140]. Bivalves, fishes and macro-sized vertebrates, several zooplankton species including *Neocalanus cristatus, Calanusfinmarchius,* and *Euphausia pacifica* have been shown to ingest wide range of MP/NPs within 0.5 nm–816 μM [139]. There are few methods for detecting NPs in biological tissues due to their small sizes, and therefore, there is limited research on the distribution, composition, and impact of NPs on the environment and organisms. More research is needed to investigate the effects of lower doses and long-term exposure to NPs in the environment. The route of entry of NPs into the food chain, its accumulation, and interaction in the environment needs to be studied [143, 144]. More research should be directed towards the use of other types of NP materials such as polyethylene terephthalate (PET), polyethylene (PE), polyvinyl chloride (PVC), polypropylene (PP) for a more comprehensive understanding of the risk and toxic effects of these materials.

## 6 Impact of nanoplastics on human health

The available evidence shows that these nanoparticles can find their way along the food chain and transfer high up to the trophic level or even along the human food chain via several pathways such as animal feed or sea salt [145]. It is vital and of great interest for further studies on the transport, absorption, and toxic effect of these particles in the body. Recent research on the harmful effects of NPs has primarily focused on their efficiency of transport and absorption in the intestine and their accumulation in the tissues of numerous model animals stating that the gastrointestinal tract, skin contact, lungs are the major human exposure pathways [24][]. NPs can reach the fish brain; however, there is only a limited amount of information about the number of particles that reach the fish brain and their potential neurotoxicity [146]. Future research could be undertaken to evaluate the potential accumulation or similar effect on humans. There is also limited information on the human health risks of NPs.

The small size of NPs (*<*1 μm) enables them to be easily consumed [147]. Little attention has been given to NPs (*<*100 nm) [148], probably because they transverse through the biological membranes and affect some cell activities, including blood cells and photosynthesis [148]. Nanoplastics have a unique obstacle in their capacity to bind cells and the gut. Recent study the planktonic filter feeder *Daphnia magna* to track the uptake route and target tissue of PSNPs using a fluorescent PSNPs (25 nm) to monitor its accumulation in both adult animals and embryos in the open brood pouch [149]. In the early stages of embryonic development, when the embryo is still covered by a chorion and before the start of organogenesis, PSNP accumulates in or on lipophilic cells, according to a time series of embryonic growth within the brood pouch [149]. In contrast, adults' gut epithelium and lipid droplets did not contain any PSNP particles [149]. In a follow up study, they showed that PSNPs caused a significant of disruption glucose metabolism, alteration in cortisol levels with a potential link to behavioral changes in larval zebrafish [150].

Following translocation, NPs can pass through the intestinal barrier and circulatory systems, depending on their surface load and size. Human (*in vitro*) and rodent (*in vivo*) bioavailability of polystyrene nanoparticles ranged from 0.2 % to 2 %. Different surface compositions and NP sizes were tested in various intestinal models (from 1.5 % - 10 %) using different polystyrene particles of 50 nm–500 nm [151]. Surprisingly, oral exposure to 50 nm polystyrene particles increased iron absorption *in vitro*, suggesting that NP exposure changes the intestinal epithelial barrier properties [152]. Polystyrene NPs influenced the viability of human stomach cancer cells, as well as inflammatory genetic expressions, such as IL-6, IL-8, and two primary gastric cytokines of stomach adenocarcinoma cells [153]. The evidence from *in vitro* research using mammalian cells is currently scarce. Nanoparticles can pass the intestinal barrier depending on their surface charges and size [154]. Previous research suggests that absorbed microplastics are barely absorbed by spleen excision, both *in vitro* and *in vivo* [155]. When NPs and microplastics enter the bloodstream, they can harm or disrupt the reproductive and nervous systems. A few research have focused on the potential bioaccumulative effects of nanoplastics. Rossi et al. [156] discovered that nano-sized polystyrene particles can easily penetrate fat membranes and that polystyrene chains caused many changes to the membrane structure inside the membrane core, significantly reducing membrane spreading and thus affecting cell function, using coarse-grained molecular simulations.

A recent study has shown that inhaled nanoparticle-containing aerosol and subsequent diffusion into the blood circulation could disturb the human respiratory system [157]. For example, according to Vianello et al. [158], humans can inhale up to 272 particles of airborne plastic each day from indoor air. Similarly, Enyoh et al. [159] demonstrated that particles with a diameter of fewer than 2.5 μm might remain in the lungs and pass through the respiratory barrier. It has also been reported that only positively charged PS-nanoparticle could induce cytotoxicity in a dose-dependent manner [160]. PS-NPs could also be internalized via non-specific phagocytosis. Higher and lower concentrations have the potential to inhibit cell viability in a dose-dependent and size-dependent manner, respectively [161]. Generally, these findings suggested that NPs could harm the human respiratory system. However, more research is needed to examine the potential health risks connected with inhaling NPs of various sizes, shapes, and concentrations. Furthermore, Campbell et al. [162] discovered that PS-NPs with diameters ranging from 20 to 200 nm might penetrate the stratum corneum to a depth of 2–3 μm. Despite this, there is still uncertainty about whether NPs could permeate the human epidermis due to the stratum corneum's ability to operate as a physical barrier [24].

Furthermore, the hydrophobic property makes it difficult for NPs to permeate through the skin in water. The use of components in personal care products, on the other hand, may help NPs diffusion. According to Jatana et al. [163] components in skincare lotions (such as urea, glycerol, and alpha hydroxyl acids) can help quantum dot nanoparticles (20.9 nm) diffuse into expurgated human skin. So yet, no research has shown that NPs diffuse directly into human skin. Because only NPs (100 nm) can diffuse into human skin, similar study efforts are urgently needed for NPs to ensure these pieces of evidence.

## 7 Remediation strategies for nanoplastic surge in Africa

Nanoplastics are water-insoluble solid polymer particles manufactured for various applications in cosmetics, clothing, construction, agriculture, packaging materials, marine, biomedical, pharmaceuticals, and personal cares industries, etc. [164]. Their unique size (<100 nm) and characteristics make them more toxic than microplastics on living tissues [165, 166]. They can easily readily pass through the cell membrane, adversely altering the brain tissue morphology, reproduction rate, liver and muscle metabolism [167, 168, 169, 170, 171]. NPs tend to be challenging to handle and remove, and studies on their removal is relatively scarce because most reports are on plastic particles with size ≥1 μm (1000 nm). However, few studies have elucidated NPs removal using physical, chemical, and biological technologies, which is discussed in this section.

### 7.1 Physical and chemical removal technologies for NPs

The environmental effects of NPs have motivated several kinds of research on various physical and chemical processes employed for its removal from water, wastewater, and other environmental samples. Murray & Örmeci [172] investigated the removal of NPs with gravity-based and mechanical separation processes on a bench-scale using synthesized (laboratory-created) NPs (<400 nm). Filtration, centrifugation, and ballast flocculation were all evaluated for their ability to remove the NPs particles. The highest NPs removal efficiency of 84 % (using 0.7 μm filters) was achieved for filtration, 94 % (at 7000 rpm) and 99 % (at 10 min) for centrifugation and 77 % with ballasted flocculation. The results revealed that NPs removal was largely influenced by pore size, centrifuge speeds, and time. Another study reported that the removal efficiency of micro-and nanoplastics (180 nm–125 μm) by coagulation/flocculation combined with sedimentation (CFS) was not sufficient to remove micro-and nanoplastics. The sedimentation rate of clean plastics was <2.0 % for all particle sizes with Al_2_(SO_4_)_3_ coagulant, even the addition of coagulant aid (Diallyldimethylammonium Chloride), achieved the highest removal rate of only 13.6 % for 45–53 μm particle sizes. However, when granular filtration was applied, the removal efficiency increased from 86.9 % to almost complete removal of 99.9 % for particles >100 μm [173]. A recent study using granular activated carbon (GAC) found that in ultrapure water, the adsorption and removal of NPs were dominated by electrostatic attractions between the positively charged polystyrene (PS) NPs and negatively charged GAC. More so, PS NPs adsorption capacity was found to increase with increasing initial NPs concentration with a maximum adsorption capacity equal to 2.20 ± 0.06 mg/g, while the removal efficiency decreased from 98 % to 26 %. When applied to natural surface water from Lake Geneva, the adsorption capacity significantly increased with increasing PS NPs concentration with a maximum adsorption capacity of 6.33 ± 0.20 mg/g. The higher removal efficiency was observed in Lake Geneva water, especially at higher NPs concentrations (from 10 to 40 mg/L), reaching 90 % of removal at 20 mg/L [174].

Various other physical and chemical remediation techniques for NPs using electrosorption [175], photocatalysis [176], biochar [177], including a schwarzite-based moving bed 3D printed water treatment system [178], have been documented. Beside the schwarzite-based moving bed 3D printed water treatment system, these techniques are inexpensive and easy to apply for large scale removal of NPs in Africa, however, the time required is a major drawback. For example, photocatalysis technique utilizes titanium oxide as a photocatalyst and solar radiation to convert the plastic material (photocatalyte) into low molecular weight intermediate products [179]. This is highly cost-effective as it uses renewable energy [180]. Since most published researches have been focused on the treatment and removal of MPs from different matrixes rather than NPs. However, studies on the elimination of NPs are still an emerging field of rapidly evolving research. Therefore, more research is necessary on other physical and chemical processes for NPs removal, such as electrooxidation, air flotation, membrane bioreactor, electrocoagulation, etc. It is noteworthy to mention that the high removal rate of NPs achieved using conventional physical and chemical methods results in a major disadvantage, which is the formation of large amounts of chemical sludge, associated with several complications such as an increase in turbidity and reduction in disposal potential [181]. This has stimulated much research into safer and eco-friendly approaches with minimum adverse effects such as bioremediation.

#### 7.1.1 Chemical treatment

Chemical treatment is one of the methods for effectively extracting MPs from various compartments. The chemical digestion of MPs can now be divided into two groups: acid and alkaline treatments. Both treatments could break down organic substances in tissues and food samples without causing plastics to break down [182]. When it comes to alkaline solutions, however, the focus is crucial for digestion. Other substances have been tested in recent years to see what benefits and drawbacks they offer. Microscopy was used to investigate the possibility of Nile Red dye for measuring MPs in bottled water. Nile Red was found to selectively adsorb MPs in water [183, 184], with propylene being the most common polymer type (54%), next in line is co-polymers, and then lubricants. Furthermore, 95% of the MPs were between 6.5 and 100 microns [185].

Microplastic research has seen a rise in the use of acids to break down organic material [186, 187]. Acid digestion, on the other hand, has several drawbacks. Although acids can dissolve polymers, between 94–98% of biogenic chemicals may be destroyed. Certain polymers have a limited acid resistance at high concentrations and temperatures and can deteriorate [188]. Acrylonitrile butadiene styrene (ABS), polyamide (PA), polyurethane (PU), polyvinyl chloride (PVC), and polymethyl methacrylate (PMMA) have all been found to be negatively affected by perchloric acid and nitric acid (70% HClO_4_ + 69% HNO_3_) suggested by the International Council for the Exploration of the Sea (ICES) [189]. Samples may be digested 26 times quicker by heating nitric acid; however, high temperatures damage weaker polymers [190]. Several microplastics isolated from personal care items were found to melt at temperatures surpassing 60 °C in boiling experiments [190]. In addition, HCl is not advised since it doesn't completely remove all organic materials, and when employed at concentrations with high digesting effectiveness, 37 % at 25 °C, it melts polyethylene terephthalate (PET). The ICES combination damaged other polymers' structures, which comprise 69% HNO_3_ and 70% HClO_4._ They caused full decomposition of polymers such as black tire rubber elastomer, polyurethane (PU), and polyamide (PA). After heating to 80 °C, the ICES combination [189] became more damaging. As a result, the simultaneous removal or destruction of certain microplastics is a major problem with various acid digesting procedures. Acids can destroy microplastics in environmental samples, resulting in an underestimate of their presence. As acidic digestion affects several polymers, it should only be done with extreme caution when other approaches fail [191].

Potassium hydroxide (KOH) and sodium hydroxide (NaOH) are commonly used in the alkaline digestion procedure. When it comes to biomaterials, sodium hydroxide has a high digesting efficiency. However, some polymers are destroyed, such as nylon strands that are severely damaged, polyethylene that melds, and unplasticized polyvinyl chloride (uPVC) particles that turn yellow [192]. With the right concentration of KOH, biological materials can be digested successfully. In fish tissues, Karami et al. (2017) investigated the digesting efficiency of acids (HCl or HNO_3_); concentrated and diluted (5%), oxidants (NaClO or H_2_O_2_), alkalis (NaOH or KOH). The results revealed that 10% KOH at 40 °*C didn*'t destroy the polymer and was by far the most efficient.

#### 7.1.2 Fenton's reagent (H_2_O_2_ with Fe^2+^)

Wet peroxide oxidation (WPO) is an oxidative digesting process that may be performed on its own, employing just H_2_O_2_ as the sole oxidant [193]. Plastic particles can be damaged if the reaction is carried out at temperatures above those required [194, 195]. When conducting WPO, reducing the reactive temperature by using an iron catalyst (Fe^2+^) is possible. When H_2_O_2_ is decomposed using Fenton's reagent, it produces hydroxyl and hydroperoxide radicals on the spot. Low-temperature processing maintains weaker polymers, allowing for more precise data collection. Complex and organic-rich materials have been proven to benefit from this approach, despite its complexity. When compared to other procedures, it is less expensive and takes less time to prepare samples, as well as a useful tool for processing big samples that more conventional methods cannot handle. As a pre-treatment for focal plane array (FPA)-FTIR analysis [66], Fenton's method may be used to extract microplastic from organic-rich materials, such as wastewater [196], sediments [197], and biota [198], as it does not affect the microplastics' surface chemistry or particle size [194].

Neither Fenton's reagent nor WPO is without flaws, though. Fenton's reagent had a considerable influence on several microbeads examined in evaluating chemical digesting procedures [190]. Microbeads lose part of their properties when heated to a temperature of 60 °C; hence an ice bath must be used to keep a temperature below this key threshold throughout the process [190]. It is necessary to utilize an ice bath to keep the temperature below a key level to prevent the loss of microplastics. Polyethylene (PE) and polyamide (PA) have also become discolored due to Fenton's [199]. Visual detection of microplastics may be hampered by microplastics that are discolored.

#### 7.1.3 Density separation

In coastal beaches and sediments worldwide, there have been detections of microplastics [200]. Thompson et al. [201] devised the extraction technique that has gotten adopted by many researchers. To separate sediment from microplastic particles, this approach, which is now the most extensively utilized [202], depends on the density of a concentrated NaCl solution to be employed (1.2 kg L^−1^). Because of the low density of the microparticles, they float to the surface of the sediment sample when this salt solution is introduced. The greater the density of the salt solution employed, the greater the number of different types of microplastic polymers that may be removed. However, it should be noted that this approach is only efficient for polymers with densities lower than the saturated saline concentration, which is around 1.2 g cm^−3^, and that it is not suited for the extraction of high-density polymers. Plastics with densities ranging from 1.14 to 1.56 g cm^−3^ (polyvinyl chloride) and 1.32–1.41 g cm^−3^ (polyethylene terephthalate) will not float when mixed with the NaCl solution [200]. This may underestimate the quantity of the high-density microplastics from the sample matrices. Sodium chloride is an inexpensive, readily available, and ecologically friendly chemical.

Sodium polytungstate (Na_6_(H_2_W_12_O_40_)), sodium iodide (NaI), and zinc chloride (ZnCl_2_) solutions all have densities ranging from 1.6–1.8 g cm^−3^ [203]. The removal efficiency of high-density plastic polymers can be improved by using these agents, which are viewed as viable alternatives to saturated NaCl solution in some cases. Using NaI and sodium polytungstate solution to remove microplastics has been successful, although they are more expensive than the other salts [65]. Even though the ZnCl_2_ solution was employed for the microplastic separation, it contains heavy metals and therefore poses a concern to the ecological environment. As a result, recycling and reusing ZnCl_2_ solutions should be done with caution to reduce environmental pollution. Although density separation has many advantages, it also has some drawbacks. Understanding the design and sample types can help determine whether density separations should be used and, if so, what sort of separation is the most appropriate for the situation. The environmental matrix may indicate the possibility of microplastics being lost during the density separation process [191].

### 7.2 Biological removal technologies for NPs

One aspect of biological remediation for plastics involves the use of biodegradable materials (bio-based polymers obtained from renewable starting materials, such as starch, cellulose, lignin, and bioethanol) and most of which are biodegradable (i.e., polylactitol and biodegradable polyethylene) [204]. Cellulose fibers extracted from plantain pith and modified with polyethylenimine (PEI) were developed as an efficient adsorbent for three common plastic nanoparticles. High adsorption and 98 % removal efficiencies of polymer nanoparticles were obtained within 30 min, suggesting that this renewable adsorbent is a promising material for a wide range of applications owing to its biodegradability, easy accessibility, and high extraction efficiencies [205].

Another aspect of biological NPs removal is bioremediation, which involves using microorganisms such as bacteria and fungi or enzymatic hydrolysis. Microbial degradation of NPs is a promising approach gaining increasing attention. Its slow pace, incomplete mineralization, and novel degradation mechanism make this technology the focus of recent research [206]. Biodegradation of NPs usually involves the release of certain extracellular enzymes (such as hydrolases) by microorganisms, which transforms complex NP polymers into low molecular forms through biodeterioration, bio-fragmentation, biosynthesis, and mineralization [166, 207]. This process is influenced by biotic (metabolic activity, the release of acids, enzymatic activity) [208, 209], abiotic factors (surface morphology, topography, surface hydrophobicity, electric charge distribution), and other environmental conditions (such as temperature, pH, salinity, oxygen level) [207, 210].

The surface area to volume ratio of NPs provides a large area for microbial colonization and their possible degradation. Their biodegradation rate depends on their molecular weight, crystal structure, organic functional groups, and additives [211]. Determination of the extent of biodegradation can be achieved through the quantification of aerobic (CO_2_) and anaerobic (CH_4_) emission during mineralization of NPs [212].

In the last decade, multiple efforts have been made to identify and isolate microorganisms capable of utilizing and degrading synthetic polymers, and bacterial taxa such as *Arcobacter*, *Vibrio,* and *Campylobacter* have been reported to degrade NPs during the treatment of wastewater [213]. Balasubramanian et al. [214] identified two potential HDPE degrading marine bacteria such as *Arthrobacter* sp. and *Pseudomonas* sp. Sivan [215] found that *Brevibacillus* spp. and *Bacillus* spp were capable of degrading polyethylene. Yoshida et al. (2016) discovered a new bacterium, *Ideonella sakaiensis* 201-F6, with an unusual ability to utilize PET as its major carbon and energy source for growth. This new species, *Ideonella sakaiensis*, could break down the plastics using two enzymes to hydrolyze PET and a primary reaction intermediate, eventually yielding basic building blocks for its growth. Species belonging to Pseudomonas, Streptomyces, Bacillus, Corynebacterium, Arthrobacter, Micrococcus, and Rhodococcus are most often reported as prominent microbial agents used for biodegradation, the genus Bacillus having the highest capacity of biodegradation compared to the other genera [216, 217, 218]. Bacteria can degrade NPs in contaminated water by producing certain enzymes (PETase and MHETase), preceded by the formation of biofilms on the surface of NPs, resulting in its fragmentation into smaller particles and then biodegradation [166].

Liu et al. [219] reported that most NPs eliminated during wastewater treatments retain in the sludge. Some authors observed a higher occurrence of NPs in the sludge than in the wastewater [220, 221]. Bacterial strains such as *Bacillus* sp, *Paenibacillus* sp., *Ideonella sakaiensis,* and Rhodococcus have been reported to successfully degrade plastic polymers from sludge and municipal solid wastes [180, 222, 223].

A more recent greener and effective strategy that has generated considerable interest is the enzymatic degradation of plastics. Diverse microbial enzymes, such as laccases, cutinases, lipases, horseradish peroxide, esterases carboxylesterases, etc., have been reported to alter and/or degrade plastic fragments [224, 225]. The degradation of polyurethane microplastics was initiated by polymer binding peptides, such as anchor peptides, which serve as synthetic polymers' binding tools. Moreover, enzymes (lipase, esterases, and hydrolases) from fungi species such as *Fusarium solani*, *Aspergillus fumigates*, *Candida ethanolica*, *Candida rugosa*, and *Penicillium chrysogenum*, have also been reported to degrade plastic polymers [226, 227, 228]. Interestingly, immobilization of some oxidative enzymes has shown high stability, durability, reusability, and cost-effectiveness compared to free enzymes [225]. These remarkable results portray enzyme remediation as a promising technology for NPs biodegradation in diverse applications.

## 8 Problems facing NPs management in Africa

### 8.1 Inconsistencies in the enforcement of waste management policies

Nanoplastics (NPs) are formed when plastic wastes are broken down, and any regulation or policy enacted to limit the use, disposal, or impact of plastic wastes will necessarily limit and lessen the impact of NPs on the environment. Many African countries have passed legislation dealing with environmental regulation, management, and protection. These include environmental protection and pollution laws, waste management laws, notably plastic waste. In certain African countries, these laws, regulations, or policies do not exist. In countries where these regulatory laws already exist, their implementation and enforcement are woefully ineffective. The African continent has the highest number of countries that has enacted legislation to control the use of plastic bags. Some of these countries include Mali, Kenya, Zimbabwe, Guinea-Bissau, Ethiopia, Eritrea, Tanzania, Chad, Cameroon, Mauritania, Morocco, Niger, Somalia, Rwanda, Tunisia, Mozambique, Botswana, and South Africa [229]. Rwanda has led Africa's fight against plastic bags. Other countries have also moved to either ban or limit their usage. Eritrea in 2005, banned plastic bags outright. Similarly, the Republic of Congo has banned the production, import, sale and use of plastic bags in a move to fight environmental pollution. The government adopted a decree prohibits the use of plastic bags to pack food, groceries, water and other beverages [230]. Other successful cases have been observed in Eritrea, where plastic bags have been replaced by cotton bags, and in Senegal, where recycling system has been implemented for the recovery of plastic products [229]. Weak or poor plastic bag ban enforcement has been detected in DRC, Cote d’Ivoire, Ethiopia, Mali, Malawi, Niger, Morocco, and Tanzania [229].

### 8.2 Poor waste management

Although Africa has been quickly developing and consuming enormous volumes of plastic products as part of its economic development, no viable plastic waste management procedures exist [231]. As a result, NP contamination threatens Africa's terrestrial, freshwater, and marine environments. In most industrialized countries, plastic garbage is frequently segregated from other debris at the source before being disposed of [232]. Recycling, recovering, and reusing are just a few of the sustainable solutions for managing plastic trash. This is not the situation in many African countries and can be the major cause of plastic pollution in Africa [231].

### 8.3 Pervasive corruption

Corruption is a pervasive and fundamental threat in most African countries, and it is widely viewed as one of the major impediments to Africa's political, economic, social, and scientific growth [233, 234]. No matter how well-intentioned, regulations will be rendered useless if the persons charged with enforcing them are corrupt or if the means of implementing them are ineffective. According to findings from studies undertaken in several African countries, the corruptibility of waste management enforcement authorities corrodes citizens' desire to obey waste disposal legislation [235]. Corruption has profoundly impacted people's psyches, thwarting any progressive attitudes toward supporting the legislation enacted by constituted authorities [236]. This pervasive corruption and lack of true dedication on the part of the government officials and their agencies have a severe impact on the control of plastic and micro/nanoplastic pollution [237].

### 8.4 Poor environmental values, risk perception, and education

Various research has shown a direct correlation between people's values, behaviors, and environmental attitudes, regardless of race, color, or societal level [238, 239]. For effective control of plastic wastes, the entire populace must be adequately involved as this gives them a sense of belonging and to be committed to the successful implementation of plastic waste legislation. Plastics bans are also criticized by many people in different African countries as they create unemployment in the plastic industry and further marginalize indigent consumers since they are very sensitive to slight changes in prices [240]. Plastic waste ban resistance has been observed in counties like Zimbabwe, Benin, Ethiopia, Ghana, Mali, and Uganda [241]. The plastic ban resistance in these countries may be attributed to Poor environmental values, risk perception, and lack of awareness of the dangers of plastic wastes. Therefore, effective legislation is hinged on consistent enforcement and educating the public to attain environmental buy-in [241]. Thus, governments and local authorities should do extensive public awareness and educative programmes to enlighten consumers about the detrimental effects of plastics and the need to use substitutes such as other biodegradable bags as they are environmentally friendly.

### 8.5 Lack of modern research facilities

On the African continent, there is a significant knowledge gap about environmental pollution. Most scientific studies on NPs pollution require sophisticated scientific equipment, which are scarce in most African countries. One of the reasons for the scarcity of data on NPs pollution in Africa could be because of this. The majority of African scientists and researchers rely on partners from developed-country facilities to complete their analyses and other experimental work. Several exchange programs have allowed some African researchers to visit well-equipped NPs laboratories. However, because the requisite analytical facilities are missing in Africa, African researchers on return from such exchange programs are constrained in their capacity to transfer their newly gained knowledge and abilities. Overall, Africa's ability to collect comparable long-term data for monitoring, risk assessment, and successful implementation of intervention options is harmed.

## 9 Conclusion, recommendations and future prospects

Plastics have become one of the most prevalent and harmful environmental contaminants, pervading all aquatic and terrestrial environments. Plastics' increasing pervasiveness in the environment and their severe potential ecological and toxicological repercussions, both known and unknown, has piqued the curiosity and attention of scientists and the general public and policymakers. Africa is a developing continent with a great demand for plastic products due to their low cost and easy availability. Despite the fact that Africa has been quickly producing and consuming enormous amounts of plastic products as part of its economic development in recent years, there is a lack of proper plastic waste management practices and public awareness of plastic pollution. Given the ever-increasing contamination of many environmental matrices and ecosystems in Africa by micro- and nanoplastics, immediate action is required to address the situation. We propose different ways by which this can be achieved. The creation and strict execution of policies aiming to build adequate waste management systems and combat plastic pollution are the first steps in decreasing the threat of nanoplastic pollution in Africa (Table 3). Existing rules and regulations relating to plastic manufacture and trash disposal should be gradually re-evaluated and reviewed in order to make them more effective, amenable, and attainable. These policies and regulations should encompass holistic waste management strategies and provide incentives to encourage improvements, such as the design and development of biologically and environmentally friendly plastic materials. Recycling, recovering, and reusing are just a few of the sustainable solutions for managing plastic trash. Besides, the availability of credible data on the occurrence, distribution, transportation, and environmental implications of NPs is required for effective policy design. The various African nations should encourage and fund research to improve knowledge about plastic contamination in the environment at all levels. As a result, the government will better formulate policies and regulations to promote better plastic waste management across the continent (Table 3). Furthermore, innovative solutions, such as the advent of biodegradable polymers (bioplastics) and other alternatives, are also needed, particularly for packaging. Bioplastics have the potential to help reduce pollution caused by rising global demand for and dependency on plastics. Despite the inherent benefits of employing biodegradable polymers, the commercialization of biodegradable plastics in Africa has been a resounding failure. This is mostly owing to bioplastics' higher production costs compared to their petrochemical-based counterparts.

We strongly recommend that plastic education and awareness be included in school curricula across Africa's educational system. This would be extremely beneficial in terms of lowering plastic pollution and fostering enhanced waste management. This curriculum policy can be constructed in such a way that it promotes awareness, a fundamental grasp of the repercussions of plastic waste and pollution, and instills civic responsibility in citizens. These, if properly followed, will protect the environment while also driving behavioral and social changes for improved environmental ethics.

Finance is another pressing concern in Africa's plastic waste management. Therefore, imposing a fee on plastic importation and production is one strategy to help solve this problem. Plastic manufacturing is a massive and very profitable industry. The cash generated by taxes on plastic importation and production would go a long way towards funding waste plastic management, providing a good income/incentive for waste plastic collectors, and purchasing machinery and other materials required for waste plastic recycling.

In summary, this article reviewed the current research on the production and consumption of plastics in Africa and the effects of nanoplastics in different environmental compartments (terrestrial, aquatic, and atmospheric). It explained the mechanisms of potential toxicity of NPs polymers and chemicals/additives on living organisms and provided the challenges facing NPs waste management. The studies/researches on NPs pollution have received global attention, and substantial progress has been recorded in some aspects. The study of NPs pollution is an emerging field, and there are still many emerging factors that need to be explored, investigated, and unraveled in the future. However, a few points that need to be noted include:

• Many studies on the impact of NPs on aquatic environments (particularly marine habitats) and ecosystems have been conducted. However, research on the effects of NPs on terrestrial and atmospheric habitats is sparse. As a result, future research into the distribution and impact of NPs in terrestrial and atmospheric ecosystems is required.
• NPs are widely distributed in many situations, as previously mentioned, and NPs from different environments migrate to one other. To more accurately estimate the impact of NPs pollution on the environment, future NPs research must be conducted on a continental or global scale, with each environment being studied separately, taking into account its unique characteristics.
• NPs have been shown to have toxicological effects in marine and aquatic creatures in numerous studies. However, more quantitative data is needed to assess the precise toxicological impact of NPs in these aquatic creatures and a variety of different terrestrial organisms and the impact of these toxicological effects on the ecosystem.
• Studies have suggested that NPs interact with, bind or absorb other pollutants in the environments, thereby transforming their overall toxicity and bioavailability. Therefore, more studies are required to help deepen our understanding of the mechanisms behind this toxicological interaction and how these interaction impacts the ecosystem.
• Recent studies discovered the presence of NPs in human stool samples, suggesting that humans are exposed to NPs through foods and/or drinks. However, the effect of NPs on human health is scarcely researched. Therefore, we suggest that more studies are necessary to provide a comprehensive understanding of NPs pollution in Africa, to provide a better basis for the subsequent pollution management and control in the continent.

## Declarations

### Author contribution statement

All authors listed have significantly contributed to the development and the writing of this article.

### Funding statement

This research did not receive any specific grant from funding agencies in the public, commercial, or not-for-profit sectors.

### Data availability statement

No data was used for the research described in the article.

### Declaration of interest's statement

The authors declare no conflict of interest.

### Additional information

No additional information is available for this paper.
